# Dyslipidemia in Pregnancy: A Systematic Review of Molecular Alterations and Clinical Implications

**DOI:** 10.3390/biomedicines12102252

**Published:** 2024-10-03

**Authors:** Agnesa Preda, Silviu-Daniel Preda, Maria Mota, Dominic Gabriel Iliescu, Lucian George Zorila, Alexandru Cristian Comanescu, Adina Mitrea, Diana Clenciu, Eugen Mota, Ionela Mihaela Vladu

**Affiliations:** 1 Department of Obstetrics and Gynecology, Clinical County Emergency Hospital Craiova, 200642 Craiova, Romania; apcela@yahoo.com (A.P.); dominic.iliescu@yahoo.com (D.G.I.); zorilalucian@gmail.com (L.G.Z.);; 2 Department of Surgery, University of Medicine and Pharmacy of Craiova, 200349 Craiova, Romania; sdpreda@gmail.com; 3 Department of Surgery, Clinical County Emergency Hospital Craiova, 200642 Craiova, Romania; 4 Doctoral School, Faculty of Medicine, University of Medicine and Pharmacy of Craiova, 200349 Craiova, Romania; mmota53@yahoo.com (M.M.); eugenmota@yahoo.com (E.M.); 5 Department of Obstetrics and Gynecology, University of Medicine and Pharmacy Craiova, 200349 Craiova, Romania; 6 Department of Diabetes, Nutrition and Metabolic Diseases, Faculty of Medicine, University of Medicine and Pharmacy of Craiova, 200349 Craiova, Romania; diana.clenciu@umfcv.ro (D.C.); ionela.vladu@umfcv.ro (I.M.V.); 7 Department of Diabetes, Nutrition and Metabolic Diseases, “Philanthropy” Clinical Municipal Hospital of Craiova, 200143 Craiova, Romania; 8 Department of Diabetes, Nutrition and Metabolic Diseases, Clinical County Emergency Hospital Craiova, 200642 Craiova, Romania

**Keywords:** dyslipidemia, fetal outcomes, maternal outcomes, molecular

## Abstract

Background: Dyslipidemia in pregnancy presents unique clinical challenges due to its effects on maternal and fetal health. This systematic review hypothesizes that molecular alterations in lipid metabolism during pregnancy contribute to adverse pregnancy outcomes and seeks to identify the clinical implications of these changes. The rationale behind this review stems from the increased risk of complications such as preeclampsia, intrauterine growth restriction, and acute pancreatitis associated with dyslipidemia in pregnancy. The primary objective is to examine the interplay between lipid metabolism and pregnancy outcomes. Methods: To achieve this, a systematic review following PRISMA guidelines was conducted, with a comprehensive search of the PubMed database covering articles from January 2014 to June 2024. Inclusion criteria focused on studies assessing molecular alterations and clinical outcomes of dyslipidemia in pregnancy, while case reports and relevant clinical trials were analyzed to evaluate both maternal and fetal outcomes. A total of 12 studies were included in the final analysis. Results: This study provided evidence of the need for early detection and management strategies to reduce risks. The outcomes revealed significant associations between dyslipidemia and adverse maternal outcomes such as preeclampsia, gestational diabetes, and pancreatitis, as well as fetal outcomes like preterm birth and fetal distress. Conclusions: Early lipid monitoring and intervention are crucial in mitigating these risks and suggests that a multidisciplinary approach is necessary to improve maternal and fetal health in pregnancies complicated by dyslipidemia.

## 1. Introduction

Dyslipidemia, characterized by abnormally elevated levels of lipids in the blood, can significantly affect pregnancy outcomes and maternal health [1]. During gestation, physiological changes in lipid metabolism occur, which can result in complex patterns that allow dyslipidemia to affect both the mother and the developing fetus [1,2]. These lipid-related abnormalities have long been considered physiological with no significant clinical relevance during pregnancy, and thus, lipids and lipoproteins are often not routinely evaluated [3,4]. However, emerging evidence suggests that these lipid disorders may have important implications for both maternal and fetal outcomes, including the development of cardiovascular diseases (CVDs) later in life [4].

Elevated cholesterol and triglyceride levels during pregnancy are associated with maternal complications, such as preeclampsia (PE), gestational diabetes mellitus (GDM), and placental abruption [3,5,6,7,8,9]. Preeclampsia, for instance, has been linked to endothelial dysfunction caused by increased cholesterol levels, which raises the risk of this life-threatening condition [3]. Moreover, the coexistence of insulin resistance with high cholesterol and triglyceride levels increases the risk of developing GDM, while elevated lipid levels may also interfere with placental function, increasing the chances of placental abruption [6,9]. These complications emphasize the need for early identification and careful monitoring of dyslipidemia during pregnancy.

The risks associated with dyslipidemia extend to the fetus as well. Elevated maternal lipid levels can lead to fetal growth restriction, preterm birth, and fetal distress [1,10,11,12,13]. Fetal growth restriction occurs when maternal lipid metabolism affects the efficiency of nutrient delivery across the placenta, restricting normal fetal development [1]. Furthermore, conditions like PE and GDM, often necessitating early delivery, are associated with preterm births [10,11]. Dyslipidemia also increases the risk of intrauterine growth restriction (IUGR), placing the fetus at heightened risk for distress due to impaired blood flow through the placenta [13].

Hypertriglyceridemia during pregnancy presents a particularly severe challenge, as it can lead to life-threatening maternal complications such as acute pancreatitis and fatty liver disease, which further exacerbate risks to both the mother and fetus [12,13]. The altered lipid metabolism in pregnancy exacerbates pre-existing hypertriglyceridemia, significantly raising the risk of adverse outcomes [12]. Timely detection and appropriate management, such as dietary adjustments or pharmacotherapy, are essential to mitigate these risks.

The long-term health of both the mother and child is also at risk due to maternal cardiometabolic dysfunction during pregnancy. Research suggests that untreated dyslipidemia during pregnancy could accelerate the progression of atherosclerosis in both the mother and child, increasing the likelihood of CVD development later in life [14]. Addressing these lipid abnormalities early in pregnancy through regular screening and intervention could have a profound impact on reducing the burden of future cardiovascular diseases.

This review aims to emphasize the need for screening and managing dyslipidemia in pregnant women due to its consequences on maternal and fetal outcomes. By understanding the underlying mechanisms and identifying risk factors, it is possible to guide therapeutic interventions and improve both short-term pregnancy outcomes and long-term cardiovascular health. While several reviews have explored common associations between dyslipidemia and pregnancy complications, our systematic review expands on previous work by analyzing rare case reports of severe dyslipidemia, particularly focusing on cases with acute maternal and fetal complications such as pancreatitis and severe preeclampsia [1,15,16]. This focus on rare presentations, alongside cross-sectional data, helps to provide a comprehensive view that mitigates bias and strengthens the overall evidence base.

## 2. Materials and Methods

We intended to maintain a rigorous and transparent process in this systematic review; hence, the Preferred Reporting Items for Systematic Reviews and Meta-Analyses (PRISMA) guidelines were followed. In finding relevant studies, the PubMed database was searched. This ensured that high-quality articles on the subject matter were included.

The study limited itself to English-language studies published between January 2014 and June 2024. This was carried out to capture current clinical practices and molecular understanding of dyslipidemia in pregnancy by choosing a ten-year period.

A search strategy consisting of several keywords was used to carry out a search on dyslipidemia in pregnancy since it is multifaceted:

*Dyslipidemia/Hyperlipidemia*: papers concerning abnormal lipid levels;
*Pregnancy*: papers specifically focusing on pregnant populations;
*Molecular Alterations*: biological changes that lead to dyslipidemia during pregnancy;
*Pregnancy Outcome*: the effects of dyslipidemia on maternal health as well as fetus outcomes.

This search approach aimed at selecting comprehensive but targeted studies addressing the clinical and molecular aspects of dyslipidemia in pregnancy and its impact on pregnancy outcomes.

From an initial pool of 579 citations, strict selection criteria were applied. There was title and abstract screening for relevance purposes before full-text reviews could be conducted so that only those articles meeting the predetermined criteria were included. Ultimately, 12 studies were identified as appropriate for use in this systematic review. Among case reports, most of these evaluations provide important insights into the clinical presentation, management, and outcomes of dyslipidemia in pregnancy. More importantly, this review emphasizes the need to distinguish between physiological and pathological dyslipidemias and to implement standardized management protocols for dyslipidemia.

Papers were scrutinized using specific inclusion criteria that involved any diagnostic accuracy or clinical utility assessment connected with dyslipidemia during pregnancy, such as studies that specifically explore hyperlipemia in pregnant women, case reports that focus on molecular changes that go with dyslipidemia during pregnancy, studies on the effect of dyslipidemia on maternal and fetal outcomes, publications that touch on management and implications of dyslipidemia in the perspective of pregnancy, studies published in peer-reviewed journals, articles written in the English language, and research conducted on human subjects. Participants were adult women. On the other hand, exclusion criteria were studies not related to dyslipidemia in pregnancy, animal studies or laboratory tests that are not connected to human conception directly, articles solely focusing on non-pregnant individuals only, articles published in any other language apart from English, research with insufficient information/data or methodology description, duplicate publications, or studies with overlapping data.

Two independent reviewers screened the titles and abstracts of identified studies, followed by a full-text review of potentially eligible articles.

The data extraction was performed using a previously defined template, with parameters that involved details about the study, number of patients, gestational age, maternal/fetal outcome, and diagnostic results. The findings were summarized by conducting data synthesis and analysis for diagnostic accuracy, clinical utility, and impact on patient management.

## 3. Results

**Figure 1:**
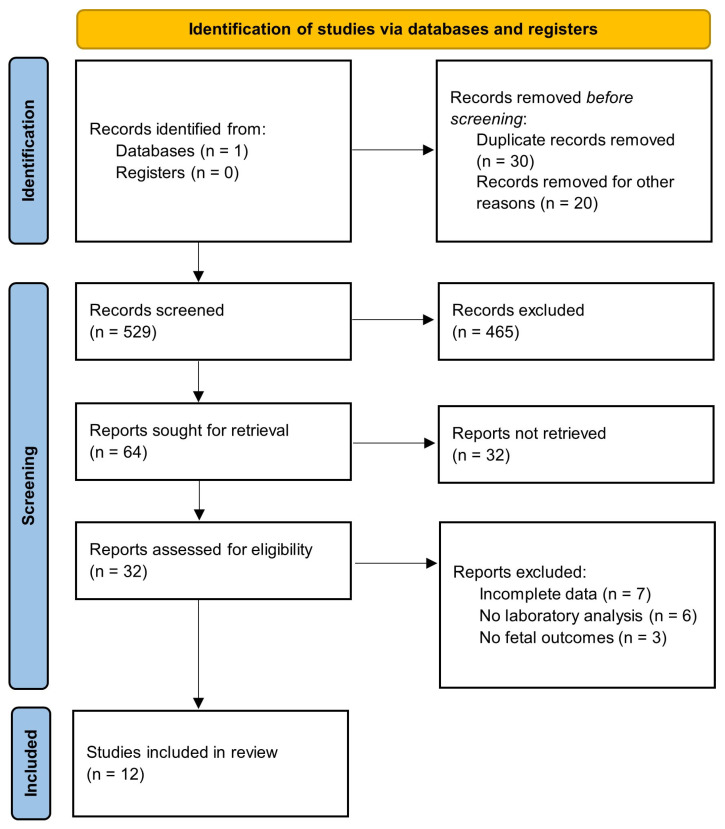
PRISMA flowchart of data acquisition.

The initial search yielded 579 results. Of these, 64 articles met the inclusion criteria based on relevance to dyslipidemia in pregnancy. Following a careful review of these articles, twelve studies were included in the final analysis (Figure 1). Those studies were all case reports. The total number of cases reported across all case reports was 12 cases.

Each report contained detailed descriptions of individual patients with dyslipidemia in pregnancy showing their clinical presentations, diagnostic processes, management techniques, and outcomes (Table 1 and Table 2). These papers were published by scholars from different countries; thus, they give a wide view of how different populations look at this problem. Cases varied greatly in terms of presentation, with some being asymptomatic while others had severe complications such as pancreatitis, preeclampsia, and gestational diabetes, among others. This entailed dietary adjustments, pharmacotherapy, or even hospitalization and intense care for those with severe conditions. In most cases, although the conditions were managed appropriately, some patients still experienced adverse events, such as preterm birth or maternal complications (Table 1).

The laboratory findings were extracted and pooled into Table 2. The discharge lab results included either the laboratory findings at discharge or after birth, depending on how it was reported by the authors.

Although physiological changes in lipid metabolism are known to occur throughout pregnancy, leading to increased lipid levels, the case reports included in this review did not track lipid changes consistently across all stages of pregnancy. Instead, lipid levels were assessed following the onset of symptoms, such as abdominal pain or acute pancreatitis, which prompted clinical investigation. Excessive lipid levels during pregnancy can have serious implications for both maternal and fetal health. The analyzed case reports highlighted maternal complications such as acute pancreatitis, preeclampsia (PE), and gestational diabetes mellitus (GDM), while fetal complications included preterm birth, fetal distress, and, in severe cases, neonatal death.

Early detection and management of dyslipidemia are essential to mitigate these risks. Regular lipid monitoring, dietary adjustments, and appropriate medication can significantly reduce complications. A multidisciplinary approach involving gynecologists, endocrinologists, and specialists in gastrointestinal diseases is recommended to optimize maternal and fetal outcomes.

Common symptoms reported in the case studies, including abdominal pain, nausea, and vomiting, are critical for timely diagnosis and management of dyslipidemia. Delayed diagnosis increases the risk of severe maternal and fetal outcomes, including poor fetal growth and long-term maternal health complications.

In women with familial hypercholesterolemia, the natural increase in lipid levels during pregnancy heightens their risk of developing complications such as PE and GDM. Insulin, commonly used to manage diabetes, has also proven effective for severe hypertriglyceridemia in non-diabetic pregnant women, as shown by Ali et al., where insulin was a potent remedy for lowering triglyceride levels in such cases.

**Figure 2:**
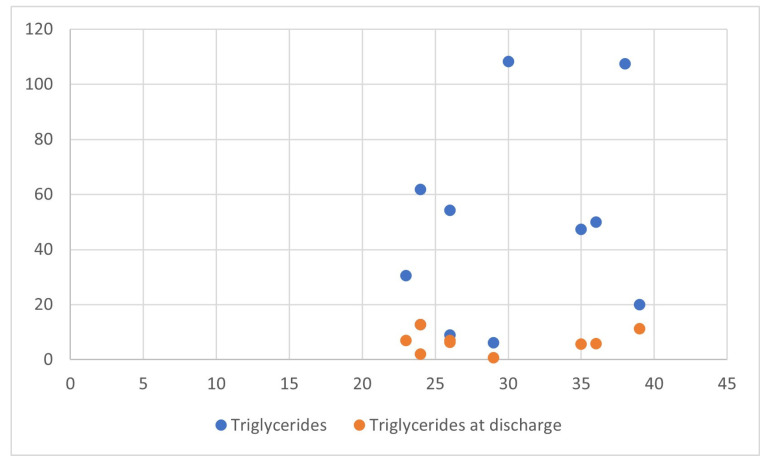
Efficacy of treatments in reducing triglyceride levels.

In the reviewed cases, lipid level changes were extreme: total cholesterol saw an average increase of 372% from baseline, HDL levels rose by 179%, LDL increased by 355%, and triglycerides had the most dramatic rise at 2326%. Interventions, including dietary changes, lipid-lowering agents, and insulin therapy, were able to reduce cholesterol levels by an average of 290% and triglycerides by 550%, demonstrating the effectiveness of these management strategies (Figure 2).

**Figure 3:**
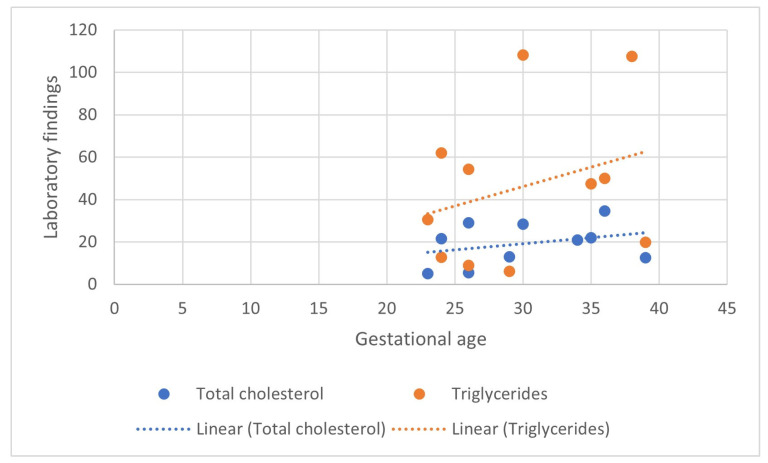
Scatterplot of cholesterol and triglyceride levels at the moment of admission in relation to the gestational age.

Most of the cases occurred in the 2nd and 3rd trimesters, where trendlines show an increase in the occurrence of both total cholesterol and triglycerides toward term (Figure 3).

Dyslipidemia-related preterm births are often due to systemic inflammation and vascular dysfunction, which impair placental function and may necessitate early delivery. These conditions are further exacerbated by coexisting GDM and PE. In severe cases, early delivery may be the preferred intervention to prevent maternal complications such as pancreatitis or severe hypertension, both of which indirectly increase the likelihood of preterm birth.

Fetal distress, characterized by abnormal heart rate patterns and reduced fetal movement, is often linked to placental insufficiency caused by elevated lipid levels. This can lead to inadequate oxygen and nutrient supply to the fetus, resulting in distress. In such cases, emergency cesarean delivery may be necessary. Dyslipidemia associated with severe PE, intrauterine growth restriction (IUGR), and premature birth increases the risk of neonatal death.

Preterm infants and those who experience fetal distress may face complications such as respiratory distress syndrome and infections, necessitating intensive neonatal care to improve survival outcomes. Regular monitoring of maternal lipid, glucose, and liver function levels, alongside fetal assessments, is crucial for detecting distress or growth abnormalities.

The review also found possible predictors for dyslipidemia: a higher maternal age (30 or older) was present in more than half of the included cases (7/12), and obesity was observed in 2 of 8 cases. A familial history of dyslipidemia was noted in 3 out of 12 cases, suggesting an underlying genetic predisposition, which may increase susceptibility to lipid abnormalities during pregnancy.

Managing dyslipidemia during pregnancy involves a combination of lifestyle modifications, including a low-fat, low-sugar diet, and the use of lipid-lowering medications. However, the use of drugs like statins is limited due to potential teratogenic effects, necessitating careful medical supervision.

Ong et al. demonstrated that a conservative approach combining a low-lipid diet and omega-3 supplementation effectively managed dyslipidemia during pregnancy [20]. This strategy helped maintain a healthy lipid profile and ensured favorable outcomes for both the mother and child.

The studies reviewed highlighted various treatments, including fibrates, statins, insulin, and plasmapheresis, though randomized controlled trials comparing these approaches have yet to be conducted. For instance, Lim et al. reported a case of acute hypertriglyceridemic pancreatitis where high doses of omega-3 fatty acids, fenofibrate, and lifestyle changes failed to reduce triglyceride levels.

The outcomes of the review are as follows:

- Maternal: Appropriate management led to favorable outcomes for many women with dyslipidemia. However, in severe cases, there was an increased risk of PE, GDM, and pancreatitis.
- Fetal: Effective management of maternal lipid levels was correlated with better fetal outcomes, reducing incidences of preterm birth, fetal distress, and neonatal complications.

This highlights the importance of early detection and personalized management strategies in improving pregnancy outcomes for women with dyslipidemia.

## 4. Discussion

The review highlights the importance of diagnosing abnormal dyslipidemia in pregnancy. Although increased levels of lipids are normal, they can be harmful when the level is very high.

The distinction between physiological and pathological dyslipidemia is crucial [8]. Elevation in lipid levels during pregnancy is usually moderate and necessary, but excessive elevations can be dangerous and harmful [8]. Detecting when dyslipidemia turns to being pathological is vital for avoiding the risk of complications.

These findings indicate that not all pregnant women need to have routine checks for dyslipidemia; however, those presenting with a history of lipid disorders or related symptoms should be closely observed.

There should be standardized guidelines on the management of dyslipidemia in pregnancy to ensure maternal and fetal safety. The case reports demonstrate various management approaches, but an integrated method would benefit clinical practice.

The small number of cases and type of cases presented underscore the necessity for broader studies, including observational and interventional studies, to better understand the implications of dyslipidemia during gestation.

Pregnancy-induced dyslipidemia is a widely studied physiological event that occurs due to the increased metabolic demands of the fetus [28]. Although usually harmless, it must be carefully observed in some cases to avoid complications. The findings from multiple studies confirm normal trends of lipid alterations and emphasize the necessity of comprehending these changes in relation to maternal and fetal health generally.

Nausea and vomiting during pregnancy are often associated with morning sickness and hyperemesis gravidarum. However, in certain cases, these symptoms must be interpreted in a context where they can be connected to an underlying dyslipidemia. It is critical for healthcare providers to recognize potential connections to ensure timely diagnosis. Healthcare providers need to seek a full medical history with regard to the patient’s past lipid disorders, any family history of dyslipidemia, or any other conditions that might predispose one to high lipid levels. Hyperemesis can be differentiated from other causes through an extensive clinical evaluation that considers symptom duration and severity, weight loss, and dehydration signs. Lipid level tests, liver enzymes, and pancreatic enzymes are important. Increased pancreas enzymes together with higher cholesterol and triglyceride levels may show acute pancreatitis brought about by dyslipidemia. It is important to recognize and treat early most cases of severe nausea and vomiting in pregnancies resulting from dyslipidemia. Some management strategies include dietary modifications with a low-fat diet to control lipids and medication use, which is paramount in controlling lipids levels.

Acute pancreatitis is an inflammatory disease of the pancreas that can be severe. Although it is rare, acute pancreatitis during pregnancy poses significant risks to both the mother and the fetus [29,30]. One of the leading causes of acute pancreatitis in pregnant women is dyslipidemia. Triglycerides can cause extremely high levels of lipids, thereby causing severe pancreatitis [31,32]. Inflammation and necrosis are caused by a buildup of triglyceride-rich lipoproteins within the pancreas, which represents the pathophysiology of the disease. An inflammatory response occurs in pancreatic tissues because free fatty acids are released when these lipoproteins are broken down by pancreatic lipase.

There are numerous genetic disorders connected to dyslipidemia [33,34]. This impact is even more pronounced when it comes to pregnancy. During pregnancy, the physiological processes can intensify already existing genetic conditions, leading to increased lipid levels and, subsequently, higher risks for both the mother and fetus. Pregnancy, with its increased metabolic demands to support the growth and development of a fetus, can lead to changes in lipid metabolism that worsen genetic dyslipidemia.

Insulin resistance is one of the factors that link dyslipidemia with GDM. These two conditions are similar because they can lead to changes in metabolism that can affect and have negative influences on both the mother and fetus [35]. GDM and dyslipidemia often occur together due to common pathophysiological mechanisms [6]. Hyperglycemia can worsen the condition of dyslipidemia through increasing lipolysis and hepatic lipid synthesis, thus creating a metabolic dysregulation cycle [36]. The co-occurrence of GDM and dyslipidemia during pregnancy can result in several undesired outcomes and long-term risks.

Understanding and appreciating this connection is very important for the prevention and correct multidisciplinary management of complications during pregnancy and postpartum.

Insulin can be a valuable treatment for hypertriglyceridemia during pregnancy not related to diabetes because it can help to increase the breakdown of triglycerides and reduce fat burning [37,38]. It is important to carefully manage this approach to ensure the safety of the patient. Insulin therapy, together with diet modification and other supportive measures, provides effective control of hypertriglyceridemia, which reduces the chances of pancreatitis in the future as well as other severe complications, thus improving both fetal health status and maternal condition. There are more factors that should be considered when starting insulin therapy: individualized treatment with a dosage and regimen that must be personalized for individual patients; the magnitude of dyslipidemia; glucose concentration as well as overall maternal health; and monitoring of the glucose levels and lipids profiles for adjusting treatment where necessary.

These facts underline that dyslipidemia in pregnancy should be diagnosed and treated as early as possible. Preventive strategies like regular lipid checks and proper pharmacological and dietary interventions are important in minimizing dangers [39,40,41,42,43]. For the best results for both the baby and the mother, a multidisciplinary method involving obstetricians, endocrinologists, diabetologists, and gastroenterologists is necessary.

Our systematic review has several limitations. The small sample size and the review based on case reports, while valuable for calling attention to rare, unusual, and severe manifestations, may prove very restrictive. During the process of the review, we tried to use all-inclusive search strategies, make a rigorous assessment of quality, and place the findings within a broader field of evidence. By admitting such predispositions and limitations openly, we can produce an accurate and dependable synthesis of evidence presently available on dyslipidemia issues in pregnancy.

## 5. Conclusions

Dyslipidemia during pregnancy is a common but complex condition with far-reaching consequences for maternal and fetal well-being.

This systematic review highlights the significance of differentiating between physiological and pathological dyslipidemia, as well as early identification and all-inclusive care regimens. There should be additional studies to emphasize long-term effects, safe drug treatment options, and better comprehension regarding molecular mechanisms involved. Bridging these gaps can enhance healthcare provision for pregnant women with dyslipidemia.

### Future Directions

Managing dyslipidemia during pregnancy remains challenging, but several novel strategies offer promise for improved outcomes.

1. Pharmacological advances: developing safer lipid-lowering drugs for pregnant women, such as modified formulations of statins or PCSK9 inhibitors, is a key area for future research.
2. Nutraceuticals and supplements: Omega-3 fatty acids have shown potential in reducing triglycerides during pregnancy. Further studies on optimal dosages and other nutraceuticals like plant sterols may provide safer treatment alternatives.
3. Personalized lifestyle interventions: tailored nutrition and exercise programs, supported by digital health technologies, could help pregnant women better manage dyslipidemia and prevent complications.
4. Early screening and risk management: Routine screening and advanced lipid profiling during pregnancy, especially for high-risk women, can enable early detection and targeted interventions.
5. Multidisciplinary care: Collaborative care models involving obstetricians, endocrinologists, and cardiologists can ensure comprehensive management of dyslipidemia, reducing risks for both the mother and child.
6. Advances in the knowledge of molecular mechanisms of dyslipidemia in pregnancy can lead to targeted therapies as well as possible prevention strategies.

These approaches, focusing on early detection, safer treatments, and personalized care, are crucial to improving maternal and fetal health outcomes in pregnancies complicated by dyslipidemia.

## Figures and Tables

**Table 1:** Pooled general data and outcomes of included studies.

Case Study	Case Presentation	Age	GA *	Medical History	BMI *	Fetal Outcomes	Maternal Outcomes	Treatment
Cao et al. [17]	Pregnant woman admitted with epigastric pain	26	35 w	Diabetes, obesity	36.4	Good	Dyslipidemia acute pancreatitis, necessity of emergency C-section	Postpartum: lipid-lowering agents and insulin infusion, heparin, and plasmapheresis treatments
Barclay et al. [12]	Pregnant woman with a history of gestational diabetes	>30	36 w	Previous GD	ND	Good	Diabetes and high blood pressure; postpartum anemia and suspected sepsis	Aggressive dietary modification; fenofibrate was recommended but declined by the patient
Wang et al. [18]	Misdiagnosed case of glycogen storage disease type I during pregnancy	24	24 w	Chronic seizures	24.4	Good born at 37 w	C-section, final postpartum diagnosis of glycogen storage disease type I	Double filtration plasmapheresis therapy; diet therapy; and oral fenofibrate.
Shapero et al. [19]	Pregnant woman with familial hypercholesterolemia	32	29 w	Hypertension, prediabetes, heterozygous familial hyperdislipdemia, polycistic ovary syndrome, depression	ND	Good born at 37 w	Early-term induction at 37 weeks, uncomplicated vaginal delivery	Lipid apheresis during pregnancy—15 sessions; fenofibrate from 29 w; statins
Ong et al. [20]	A pregnant woman with a history of mixed familial hypertriglyceridemia following an episode of acute pancreatitis	31	26 w	Diabetes mellitus type 2, mixed familial hypertriglyceridaemia, acute pancreatitis	23.4	Good, born at 38 w	Uncomplicated vaginal birth, induction at 38 w	Lipid regulating medicine; diet therapy
Lim et al. [21]	Pregnant woman with severe epigastric pain, vomiting, and decreased fetal movements	27	23 w	Familial hypertrigliceridemia	21	Preterm birth	Acute pancreatitis; placental abruption	Plasmapheresis during pregnancy and postpartum; fenofibrate; omega-3 fatty acids; lifestyle measurements
Choi et al. [22]	Pregnant woman with severe abdominal pain	24	26 w	Previous acute pancreatitis during pregnancy	32	Good	Recurrent acute pancreatitis; emergency C-section;	Plasmapheresis during pregnancy; statins; fenofibrate;
Kasim et al. [23]	Pregnant woman with homozygous familial hypercholesterolemia	28	34 w	Severe hypercholesterolemia; hypertension; coronary artery disease	ND	Preterm birth	Drug-eluting stent angioplasty during second trimester of pregnancy; elective C-section	Statins during pregnancy—from 2nd trimester
Coronado Arroyo et al. [24]	Pregnant woman with somnolence and oppressive epigastric pain radiating to the back, which was associated with bilious emesis	38	24 w	Hypertriglyceridemia, history of acute pancreatitis	24	Preterm birth, neonatal death 7 days postpartum	Lung injury that required invasive mechanical ventilation; liver and kidney dysfunction; bacteremia due to pseudomonas aeruginosa; hepatosplenomegaly and pancreatic necrosis	Plasmapheresis; fenofibrate; pancreatic enzymes after discharge; fat-restricted diet and consuming food high in omega-3 fatty acids.
Hui et al. [25]	Pregnant woman with vaginal bleeding, Kussmaul’s breathing, and history of persistent vomiting for 1 day	37	39 w	Dyslipidemia	29.6	Fetal distress	Emergency C-section; ICU hospitalization	Fenofibrate
Ali et al. [26]	Pregnant woman with headache	31	38 w	No	25.9	Good	None	Insulin infusion; low-fat diet; omega-3 fish oil tablets postpartum
Amor et al. [27]	Pregnant woman with hyperemesis	34	30 w	No	ND	Preterm birth; Unexplained neonatal severe hypertension	Type V hypertriglyceridemia; emergency C-section; preeclampsia.	Intravenous fluids with a low-carbohydrate, low-fat diet

* GA—gestational age; BMI—body mass index.

**Table 2:** Pooled laboratory findings of included studies.

Case Study	Patient Age	Gestational Age	Lab Examination Test and Unit	Preadmission Results	Admission Results	Discharge Lab Results	Normal Range
Cao et al. [17]	26	35	Total Cholesterol (mmol/L)	ND	22	4.05	<5.17
			HDL-C (mmol/L)	ND	9.89	1.46	>1.3
			LDL-C (mmol/L)	ND	10.17	2.66	<2.6
			Triglycerides (mmol/L)	ND	26.3	5.66	<1.7
			Amylase (UI/L)	ND	147.6	47.4	30–110
Barclay et al. [12]	>30	36	Total Cholesterol (mmol/L) ND	4.1	34.6	12.6	<5.17
			HDL-C (mmol/L)	0.9	<0.08	ND	>1.3
			LDL-C (mmol/L)	3.2	24.82	ND	<2.6
			Triglycerides (mmol/L)	1.01	>50	5.74	<1.7
			Amylase (UI/L)	ND	ND	ND	30–110
Wang et al. [18]	24	24	Total Cholesterol (mmol/L)	ND	21.6	6.4	<5.17
			HDL-C (mmol/L)	ND	ND	ND	>1.3
			LDL-C (mmol/L)	ND	ND	ND	<2.6
			Triglycerides (mmol/L)	ND	61.91	12.8	<1.7
			Amylase (UI/L)	ND	ND	ND	30–110
Shapero et al. [19]	32	29	Total Cholesterol (mmol/L)	ND	12.93	3.88	<5.17
			HDL-C (mmol/L)	ND	ND	ND	>1.3
			LDL-C (mmol/L)	ND	9.05	1.63	<2.6
			Triglycerides (mmol/L)	ND	6.14	0.77	<1.7
			Amylase (UI/L)	ND	ND	ND	30–110
Ong et al. [20]	31	26	Total Cholesterol (mmol/L)	4.2	5.5	4.4	<5.17
			HDL-C (mmol/L)	ND	ND	ND	>1.3
			LDL-C (mmol/L)	ND	ND	ND	<2.6
			Triglycerides (mmol/L)	7.7	8.9	7.0	<1.7
			Amylase (UI/L)	ND	ND	ND	30–110
Lim et al. [21]	27	23	Total Cholesterol (mmol/L)	ND	5.02	ND	<5.17
			HDL-C (mmol/L)	ND	0.36	ND	>1.3
			LDL-C (mmol/L)	ND	ND	ND	<2.6
			Triglycerides (mmol/L)	ND	30.6	7	<1.7
			Amylase (UI/L)	ND	ND	ND	30–110
Choi et al. [22]	24	26	Total Cholesterol (mmol/L)	ND	28.96	ND	<5.17
			HDL-C (mmol/L)	ND	ND	ND	>1.3
			LDL-C (mmol/L)	ND	ND	ND	<2.6
			Triglycerides (mmol/L)	ND	54.29	6.27	<1.7
			Amylase (UI/L)	ND	347	60	30–110
Kasim et al. [23]	28	34	Total Cholesterol (mmol/L)	20	21	ND	<5.17
			HDL-C (mmol/L)	ND	ND	ND	>1.3
			LDL-C (mmol/L)	18	ND	ND	<2.6
			Triglycerides (mmol/L)	ND	ND	ND	<1.7
			Amylase (UI/L)	ND	ND	ND	30–110
Coronado Arroyo et al. [24]	38	24	Total Cholesterol (mmol/L)	ND	ND	ND	<5.17
			HDL-C (mmol/L)	ND	0.88	ND	>1.3
			LDL-C (mmol/L)	ND	0.72	ND	<2.6
			Triglycerides (mmol/L)	ND	12.76	1.98	<1.7
			Amylase (UI/L)	ND	365	ND	30–110
Hui et al. [25]	37	39	Total Cholesterol (mmol/L)	ND	12.53	9.86	<5.17
			HDL-C (mmol/L)	ND	0.44	ND	>1.3
			LDL-C (mmol/L)	ND	1.35	ND	<2.6
			Triglycerides (mmol/L)	4.92	19.93	11.3	<1.7
			Amylase (UI/L)	ND	314	120	30–110
Ali et al. [26]	31	38	Total Cholesterol (mmol/L)	ND	ND	ND	<5.17
			HDL-C (mmol/L)	ND	ND	ND	>1.3
			LDL-C (mmol/L)	ND	ND	ND	<2.6
			Triglycerides (mmol/L)	ND	107.5	ND	<1.7
			Amylase (UI/L)	ND	ND	ND	30–110
Amor et al. [27]	34	30	Total Cholesterol (mmol/L)	ND	28.38	ND	<5.17
			HDL-C (mmol/L)	ND	ND	ND	>1.3
			LDL-C (mmol/L)	ND	ND	ND	<2.6
			Triglycerides (mmol/L)	ND	108.3	ND	<1.7
			Amylase (UI/L)	ND	ND	ND	30–110

## Data Availability

We registered our systematic review in the OSF Registries with the following identifiers: (1) Associated project sf.io/tz8c5. (2) Internet Archive link https://archive.org/details/osf-registrations-vxbh8-v1 (accessed on 16 August 2024)—Registration DOI https://doi.org/10.17605/OSF.IO/VXBH8 (accessed on 16 August 2024).
